# Acute Iloprost Inhalation Improves Right Ventricle Function in Pulmonary Artery Hypertension: A Cardiac Magnetic Resonance Study

**DOI:** 10.3389/fphar.2018.01550

**Published:** 2019-01-21

**Authors:** Jing-Hui Li, Hong-Da Zhang, Zhen-Zhen Wang, Qing-Qing Lu, Dong Li, Tian-Yu Lian, Zi-Chao Lv, Xin Jiang, Yan Wu, Jue Ye, Shihua Zhao, Zhenwen Yang

**Affiliations:** ^1^The Key Lab of Pulmonary Vascular Medicine and FuWai Hospital, State Key Laboratory of Cardiovascular Disease, Chinese Academy of Medical Sciences and Peking Union Medical College, Beijing, China; ^2^Tianjin Medical University General Hospital, Tianjin, China

**Keywords:** iloprost, right ventricle, pulmonary artery hypertension, cardiac magnetic resonance, pulmonary vascular resistance

## Abstract

**Background:** Right ventricle (RV) function is among the most important prognostic factors for pulmonary arterial hypertension (PAH) patients. Inhaled iloprost, an inhaled member of the prostacyclin family, is effective for the treatment of severe PAH and acute RV failure. However, the acute effects of iloprost on RV physiology have not been thoroughly explored in the past.

**Materials and Methods:** This prospective study involved 69 incident PAH patients, including 23 idiopathic PAH (IPAH) patients, 26 patients with PAH associated with connective tissue disease (CTD-PAH) and 20 with PAH associated with congenital heart disease (CHD-PAH). All patients underwent both right heart catheterization and cardiac magnetic resonance imaging at baseline and 20 min after 5 μg iloprost inhalation.

**Results:** Acute iloprost inhalation reduced PVR from 13 ± 7 to 10 ± 6 Wood U (*P* < 0.001), increased RV ejection fraction (RVEF) from 31 ± 11 to 35 ± 12 % (*P* < 0.001), increased RV stroke volume from 53 ± 21 to 57 ± 22 ml (*P* < 0.001) and decreased RV end-diastolic volume from 179 ± 67 to 172 ± 69 ml (*P* < 0.001). Acute iloprost inhalation-induced RVEF improvement was correlated with the degree of PVR reduction (*P* < 0.001) in IPAH patients, but not in CTD-PAH or CHD-PAH patients.

**Conclusion:** Acute iloprost inhalation improved RVEF, RV stroke volume and decreased RV volume in IPAH and CTD-PAH patients. Iloprost-induced RVEF increase was proportional to PVR reduction in IPAH patients, but not in CTD-PAH or CHD-PAH patients.

## Introduction

Parenteral prostacyclin has been repeatedly shown to improve functional state, exercise capacity and outcome in pulmonary arterial hypertension (PAH) and is currently recommended upfront in PAH patients in WHO functional class III and may be considered in WHO functional class IV (Galiè et al., 2015). Inhaled iloprost, a member of the prostacyclin family, is effective for the treatment of severe pulmonary hypertension (Olschewski et al., 2002) and acute right ventricular (RV) failure (Martischnig et al., 2011). It is also used for acute pulmonary vasodilator testing (Jing et al., 2009). Acute iloprost inhalation could lower mean pulmonary artery pressure (Ivy et al., 2008) and pulmonary vascular resistance (PVR) (Martischnig et al., 2011). However, compared with the knowledge on pulmonary artery hemodynamic changes, RV physiological changes during acute iloprost inhalation were much less known. In 1999, McLaughlin and Rich reported on major increases in cardiac output in PAH patients treated with intravenous epoprostenol and thought that this was explained by the direct positive inotropic effects on the RV added to the decreased PVR (Rich and McLaughlin, 1999). The positive inotropic effects of iloprost have been reported in animal studies (Rex et al., 2008; Gomez-Arroyo et al., 2015).

Cardiovascular magnetic resonance imaging (CMR) is accurate and reproducible in the assessment of RV morphology and function. Many CMR studies have shown that decreased RV function and increased RV volume were predictive of poor outcome (van de Veerdonk et al., 2011, 2017; Baggen et al., 2016; Brewis et al., 2016; Vonk Noordegraaf et al., 2017). So far, we did not find any study using CMR to evaluate the acute effect of iloprost on RV.

The present study investigated the effects of acute iloprost inhalation on RV in PAH patients using right heart catheterization (RHC) and CMR. Furthermore, the relationship between PVR reduction and RV function improvement during this procedure was explored.

## Materials and Methods

### Patients

Eighty patients gave informed written consent to this study, which was approved by the University of Tianjin Institutional Review Board (Ethical No. 200820). They were referred to the Tianjin Medical University General Hospital, Tianjin, China or FuWai Hospital, Chinese Academy of Medical Science, Beijing, China for diagnostic workup of PAH. A diagnosis of PAH was established in agreement with RHC demonstrating a mean pulmonary artery pressure (mPAP) > 25 mmHg, a pulmonary artery wedge pressure (PAWP) < 15 mmHg and a PVR > 3 Wood units in the absence of identifiable causal cardiac or respiratory conditions or thromboembolism. Six patients without given contrast injection during CMR examination were excluded from this study, since they were either allergic to contrast agent or had renal insufficiency. Three more patients were excluded because their diagnosis was updated to chronic thromboembolic pulmonary hypertension (CTEPH) according to their latest exams, such as ventilation/perfusion scan and pulmonary angiography. The time interval between CMR and RHC was less than a week. RHC and CMR measurements were performed both at baseline and after iloprost inhalation (Ventavis^®^; Bayer-Schering Pharma, Berlin, Germany). Iloprost was diluted with saline to a concentration of 10 μg per milliliter, and 2 ml was added to a PARI LC STAR nebulizer (PARI GmbH, Starnberg, Germany). The nebulizer was driven by a PARI TurboBOY-N compressor (PARI GmbH, Starnberg, Germany) for 15–20 min, to achieve a total dose of 5 μg iloprost into pulmonary alveoli of the patients (Jing et al., 2009).

### CMR Acquisition

Cardiovascular magnetic resonance was performed with GE 1.5T Twin-speed Infinity with Excite II (GE Healthcare, Milwaukee, WI, United States) or 1.5T Siemens Magnetom Avanto (Siemens Medical Solutions, Erlangen, Germany). Fast imaging employing steady-state acquisition (FIESTA, GE) sequence or steady state-free precession (SSFP, Siemens) sequence was used for the retrospective ECG-synchronized cine scans of all participants. The segmented cine imaging was obtained in a stack of eight to ten contiguous short-axis slices (8 mm thickness, 2 mm gap) spanning the entire right and left ventricles from the base to the apex, during breath hold with expiration.

### Right Heart Catheterization

Right heart catheterization was performed using a multipurpose catheter (Cordis; Miami Lakes, FL, United States) for the measurements of right atrial pressure (RAP), PAP, PAWP, RV pressure. Cardiac output (CO) was calculated with the Fick principle. The patients were supine and the zero was leveled at mid-chest. Pressures were measured at end-expiration. A femoral artery catheter was also placed for the measurements of systemic arterial pressure (SAP). Blood samples were drawn from the pulmonary and systemic artery catheters for measurements of mixed venous and arterial oxygen saturations (SvO_2_ and SaO_2_, respectively). PVR was calculated as (mPAP – PAWP)/CO.

### Ventricular Function

All CMR images were transported to an AW 4.3 work station (Advantage Windows version 4.3, GE Healthcare, Milwaukee, WI, United States). End-systolic frames and end-diastolic frames were identified by the smallest and largest cavity area using the cine images. Endocardial and epicardial contours were manually traced from base to apex for eight slices. The papillary muscles and trabeculae were excluded from myocardium. End-systolic volume (ESV), end-diastolic volume (EDV), stroke volume (SV), ejection fraction (EF) and cardiac output (CO) were measured and calculated by Report Card 3.7 software using Simpson’s rule.

### Vascular Function

Phase-contrast MR sequences were used to measure the pulmonary artery flow and the AW 4.3 work station was used for analysis. CMR-derived indices of pulmonary vascular function include: (1) pulsatility, defined as the relative change in pulmonary artery lumen area (PA) during the cardiac cycle, or (PAmax-PAmin)/PAmin; (2) capacitance, defined as the change in volume per change in PA, or SV/(PAmax-PAmin); (3) stiffness index β, calculated from the formula: ln [sPAP/dPAP/PA pulsatility] (Sanz et al., 2009).

### Statistical Analysis

All statistical analyses were performed using SPSS v20 (SPSS, Chicago, IL, United States) and Graph Pad Prism 5.03 (Graph Pad Software, San Diego, CA, United States). The data were presented as mean ± SD unless explicitly stated otherwise. The quantitative characteristics between the two groups were compared by *t*-test or Mann–Whitney *U* test. Paired sample *t*-test or Wilcoxon signed-rank test were used to assess the difference between hemodynamic or CMR parameters at baseline and after iloprost inhalation. Linear regression analysis was used to examine the association between the right ventricular functional changes and PVR changes. A *P* value of < 0.05 was considered significant.

## Results

### Patient Characteristics

The mean age of the study population was 38 ± 10 years old. Fifty-three (76.8%) were female patients. There were 26 PAH patients associated with connective tissue disease (CTD-PAH), 23 idiopathic PAH (IPAH) patients and 20 PAH patients associated with congenital heart disease (CHD-PAH). Among the 26 CTD-PAH patients, 15 were associated with systemic lupus erythematosus, 7 with mixed connective tissue disease and 4 with systemic sclerosis. Among the 20 CHD-PAH patients, 10 patients were associated with atrial septal defects, 4 patients were with ventricular septal defects and 6 patients were with patent ductus arteriosus. No significant difference was found between the three groups with respect to age, gender, World Health Organization functional class (WHO-FC), heart rate at baseline, body surface area (BSA) or PVR (Table 1).

**Table 1:** Demographic characteristics.

	IPAH (*n* = 23)	CTD-PAH (*n* = 26)	CHD-PAH (*n* = 20)
Age (year)	34 ± 8	42 ± 12	38 ± 11
Gender (male/female)	6/17	5/21	5/15
Baseline BSA, m^2^	1.68 ± 0.18	1.60 ± 0.14	1.60 ± 0.19
Baseline HR, beat/min	87 ± 17	81 ± 12	82 ± 14
Baseline PVR, Wood U	14 ± 8	12 ± 6	17 ± 17
WHO-FC II	15	17	12
III	8	9	8


*IPAH: idiopathic pulmonary arterial hypertension; CTD-PAH, pulmonary arterial hypertension associated with connective tissue disease; CHD-PAH, pulmonary arterial hypertension associated with congenital heart disease; BSA, body surface area; HR, heart rate; PVR, pulmonary vascular resistance; WHO-FC: World Health Organization functional class.*

### Hemodynamic and Physiological Changes

**FIGURE 1:**
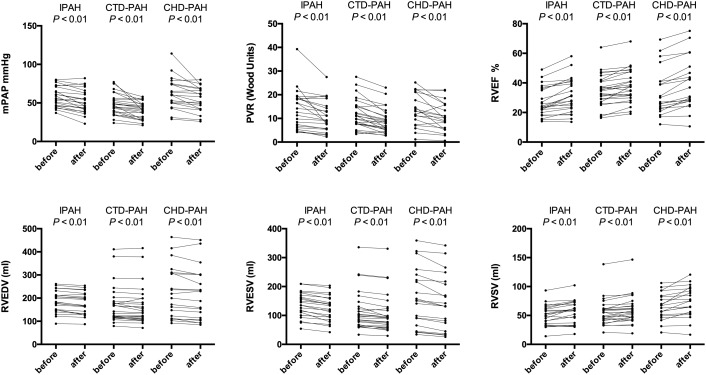
Ventricular physiology and pulmonary hemodynamics responses to acute iloprost inhalation. IPAH: idiopathic pulmonary arterial hypertension; CTD-PAH, pulmonary arterial hypertension associated with connective tissue disease; CHD-PAH, pulmonary arterial hypertension associated with congenital heart disease; PVR, pulmonary vascular resistance; mPAP, mean pulmonary artery pressure; PVR, pulmonary vascular resistance; RV, right ventricle; EDV, end-diastolic volume; ESV, end-systolic volume; EF, ejection fraction; SV, stroke volume.

**FIGURE 2:**
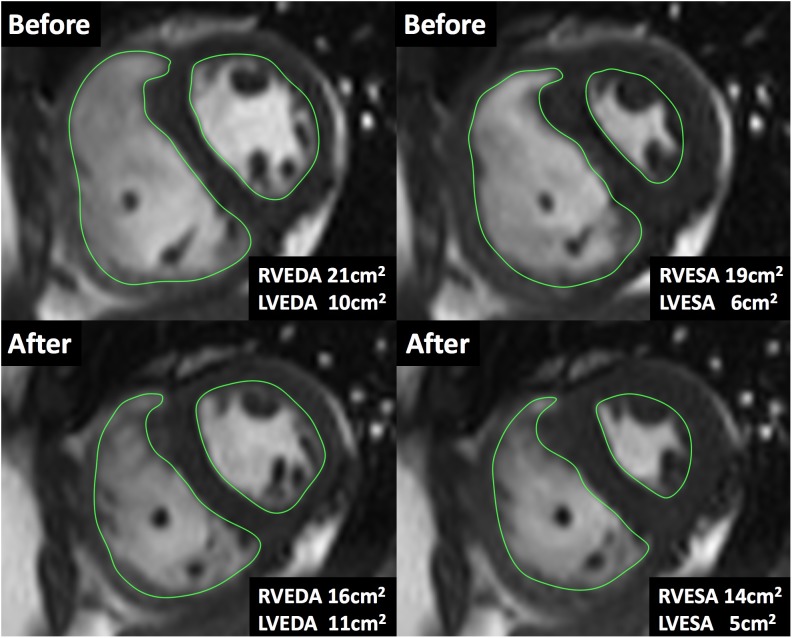
Cardiac magnetic resonance imaging during acute iloprost inhalation. A case of the short-axis view by CMR cine. The areas were measured at the same location before and after iloprost inhalation. After iloprost inhalation, RV end-diastolic area (RVEDA) was reduced from 21 to 16 cm^2^ and RV end-systolic area (RVESA) was reduced from 19 to 14 cm^2^.

The hemodynamic profile of the patients was compatible with severe PAH. In IPAH and CTD-PAH patients, inhaled iloprost was associated with average decreases in mPAP by 7 mmHg and PVR by 3 Wood units (Table 2). After acute iloprost inhalation, RV function was immediately improved, with increased RV stroke volume, RV ejection fraction (RVEF) and decreased RV volume (Figures 1, 2). Simultaneously, left ventricle (LV) systolic function also improved. Similar RV physiological changes were found in CHD-PAH (Table 2). Regarding pulmonary artery function, the only significant change was an improvement of PA capacitance (Table 3).

**Table 2:** Pulmonary hemodynamics and ventricular physiological responses to inhaled iloprost.

Variables	IPAH		*P* value	CTD-PAH		*P* value	CHD-PAH		*P* value
						
	Before	After		Before	After		Before	After	
mPAP, mmHg	58 ± 13	52 ± 15	<0.01	48 ± 13	41 ± 11	<0.01	63 ± 21	55 ± 16	0.002
PVR, Wood U	14.2 (6.8, 18.4)	9.2 (5.6, 15.2)	<0.01	11.1 (7.8, 15.2)	8.1 (5.1, 11.3)	<0.01	16.9 (7.9, 21.2)	11.7 (5.4, 16.0)	0.02
RVEDV, ml	187 ± 45	178 ± 46	<0.01	154 (118, 191)	141 (109, 199)	0.001	227 ± 114	217 ± 113	0.005
RVESV, ml	137 ± 42	123 ± 43	<0.01	98 (67, 135)	92 (63, 125)	<0.01	161 ± 101	144 ± 97	<0.01
RVSV, ml	50 ± 18	55 ± 20	<0.01	55 (43, 61)	56 (45, 67)	0.002	66 ± 23	74 ± 26	0.001
RVEF, %	27 ± 10	31 ± 12	<0.01	35 ± 11	38 ± 11	<0.01	34 ± 16	39 ± 17	<0.01
LVEF, %	59 (55, 62)	62 (55, 67)	0.002	67 (57, 76)	66 (60, 77)	0.056	56 ± 14	59 ± 14	0.01


*mPAP, mean pulmonary artery pressure; PVR, pulmonary vascular resistance; RV, right ventricle; LV, left ventricle; EDV, end-diastolic volume; ESV, end-systolic volume; EF, ejection fraction; SV, stroke volume.*

**Table 3:** Pulmonary artery morphological and functional changes after iloprost inhalation.

Parameters	Before	After
PA peak velocity (cm/s)	67 ± 25	65 ± 24
Pulsatility (%)	23 ± 16	20 ± 12
Capacitance (mm^3^/mmHg)	1.3 ± 0.8	1.6 ± 0.7^#^
Compliance (mm^2^/mmHg)	4.2 ± 2.8	4.9 ± 2.8
Stiffness index β	5.6 ± 2.8	6.1 ± 3.0


*^#^*P* value < 0.001; PA: pulmonary artery.*

### Relationship Between PVR Reduction and RV Function Improvements

To explore the relationship between RV afterload relief and RV function improvement, the correlation between PVR reduction and RVEF changes was analyzed.

**FIGURE 3:**
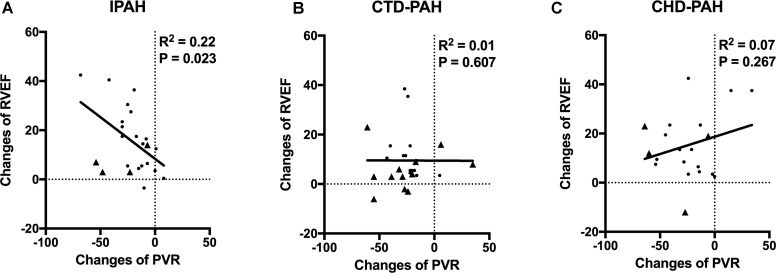
The relationships of changes of RVEF and PVR. **(A)** In IPAH patients, PVR reduction was proportional to RVEF improvement (*R*^2^ = 0.22, *P* = 0.023) after acute iloprost inhalation. Patients with positive late gadolinium enhancement in RV marked as triangle. **(B)** In CTD-PAH patients, no significant association was found between PVR and RVEF changes. More patients with positive late gadolinium enhancement in RV (triangle) in CTD-PAH compared to IPAH patients (4/23 vs. 12/26, *P* = 0.039). **(C)** No association between PVR and RVEF changes in CHD-PAH patients.

We found that RVEF improvement was proportionally associated with PVR reduction (*R*^2^ = 0.22, *P* = 0.023) in IPAH patients. In CTD-PAH patients, no significant association was found between the PVR and RVEF changes (Figure 3). This indicated that a modest reduction of PVR would not improve RVEF proportionally in most CTD-PAH patients.

Neither was the association between the PVR and RVEF changes in CHD-PAH found. The potential cause of interference here could be intra-cardiac or patent ductus arteriosus shunt.

Furthermore, we found there were more patients with positive late gadolinium enhancement (LGE) in the CTD-PAH group than in the IPAH group (12/26 vs. 4/23, *P* = 0.039).

## Discussion

The present results suggest that acutely inhaled iloprost improves RV function, in terms of RV volumes, RVEF and RV stroke volume in patients with IPAH and CTD-PAH. This knowledge is important for understanding the clinical pharmacology of iloprost for the treatment of PAH, especially on RV. This improvement of RV function would be largely explained by a reduction of afterload by iloprost. However, further analysis found PVR reduction might not guarantee RVEF improvement in some patients, especially in the CTD-PAH group. This observation could suggest more therapies on RV function in these patients.

In the present study, acute iloprost inhalation decreased PVR, increased RVEF and reduced RV volume. This was consistent with previous work on animals (Rex et al., 2008). In another short-term study, after 2 weeks of iloprost inhalation, the cardiac output and tricuspid annulus plane systolic excursion were significantly increased in a rat PAH model (Gomez-Arroyo et al., 2015). Furthermore, there was an increase in LVEF, which could not be fully explained by a decreased afterload, since mean arterial pressure was unchanged. This observation could suggest a component of prostacyclin-induced positive inotropism. The effects of prostacyclin on *in vitro* myocardial tissue preparations and animal models have been reported to be variable with no changes (Metsä-Ketelä, 1981; Wauthy et al., 2003; Holmboe et al., 2017), decreases (Karmazyn, 1986), or, more often, increases in contractility (Fassina et al., 1983; Siegl and Wenger, 1985; Kemming et al., 2005; Holmboe et al., 2013). However, based on the current data, it was not possible to determine whether the inotropic effect was direct or mediated by other mechanisms, such as systemic vasodilation or restoration of LV preload due to RV pressure reduction.

In addition, we found that RVEF improvement was proportional to PVR reduction induced by iloprost in IPAH patients but not in CTD-PAH and CHD-PAH patients. This may be explained by the fact that RV dysfunction in IPAH patients was secondary to pulmonary hypertension, while RV myocardium damage could be the result of direct infiltration of systemic inflammation in CTD-PAH patients. In addition, we observed that there were more patients with positive RV-LGE in CTD-PAH than in the IPAH group. IPAH patients with positive RV-LGE tend to have less improvement of RV function after PVR reduction by iloprost. RV-LGE could indicate myocardial cell disarray or fibrosis (Freed et al., 2012), and local LGE mass at the anterior septal insertion is reported to be associated with reduced regional longitudinal contractility at the base (Shehata et al., 2011). This could be another potential explanation for why RV function and PVR changes lost linear relationship in CTD-PAH patients. Originally, one would expect little or no response to iloprost inhalation in CHD-PAH patients. However, in this study we found mPAP was significantly reduced in some CHD-PAH patients. This is consistent with a previous study from the TOPP registry, which found 7–36% of CHD-PAH patients were acute vasodilator responders, according to different criteria (Douwes et al., 2016). Our hypothesis for this phenomenon was that vasoconstriction or spasm of pulmonary arteries existed in these patients, especially in those hospitalized patients. This could be partially corroborated by the observation that there was significant mPAP reduction in many CHD-PAH patients due to merely a sedative effect during perioperative anesthesia.

More interestingly, our present study is in line with a long-term study on the relationship between PVR and RVEF changes. Drs. van de Veerdonk and Vonk-Noordegraaf found the relative change in PVR was significantly correlated to the absolute change in RVEF after combination therapy. In contrast, they did not find a significant correlation between the change in PVR and RVEF after monotherapy (van de Veerdonk et al., 2017). The damage of linear relationships between the changes in PVR and RVEF may indicate the irreversibility of RV dysfunction, especially when treatment was insufficient. Whether the relationship between the changes of RVEF and PVR during acute iloprost inhalation could predict long-term effects of PAH-targeted therapy, remains further study.

Finally, it is of interest that the measures of PA stiffness were not affected by inhaled iloprost in the present study. This may be explained by the fact that proximal pulmonary arterial compliance as measured by CMR does not account for more than 20% of total compliance as pulmonary vessel distensibility is distributed to the entire pulmonary vascular tree (Saouti et al., 2009). Interventions that decrease PVR and increase compliance in PAH may not necessarily be associated with significant changes in proximal pulmonary arterial stiffness.

There were several limitations in this study. To capture the minor changes induced by acute iloprost inhalation, we relied on the analysis of CMR images. It is possible that the tracings of RV contour may have been different among individual operators. However, the primary data were all analyzed by two experienced radiologists and the blinded review found minimal inter-observer error on random cases. Secondly, it is always recommended to collect the CMR and RHC data simultaneously, however it is impractical in our centers. The time interval was limited to within a week in order to make sure the heart rate, hemodynamics, symptoms and treatment were stable during this time. Lastly, as this is an acute drug effect study, long-term follow-up data would be needed for enhancing clinical value.

In conclusion, the clinical importance of this study is to provide a detailed knowledge of how RV physiology is changed during acute iloprost inhalation, by combining hemodynamic assessment and CMR imaging. It was observed that RVEF improvements are proportional to PVR reduction in IPAH patients. In contrast, for CTD-PAH and CHD-PAH patients, PVR and RVEF changes are not correlated with each other. For these patients, a modest decline of PVR would mean little to RVEF improvement, thus more effective treatment may be suggested; this may be the potential clinical value of the present study.

## Author Contributions

ZY, SZ, J-HL, and H-DZ conceived and designed the study. JHL, H-DZ, Z-ZW, XJ, YW, JY, Z-CL, Q-QL, and T-YL acquired and analyzed the data. J-HL, H-DZ, DL, and YW involved administrative, technical, or material support. ZY supervised the study. All authors performed writing, review, and/or revision of the manuscript.

## Conflict of Interest Statement

The authors declare that the research was conducted in the absence of any commercial or financial relationships that could be construed as a potential conflict of interest.
